# Repercussions of neck pain on the quality of life of health professionals in Intensive Care Units

**DOI:** 10.17533/udea.iee.v42n3e06

**Published:** 2024-10-19

**Authors:** Alberto de Oliveira Redü, Daiani Modernel Xavier, Marcela Amaral Daoud, Giovana Calcagno Gomes, Eliane Raquel Rieth Bennetti, Franciele Gomes Soares, Luciano Garcia Lourenção

**Affiliations:** 1 . Physical therapist, Master. Email: betoredu@hotmail.com. Corresponding author. https://orcid.org/0000-0003-4774-3090 Universidade Federal do Rio Grande do Sul Brazil betoredu@hotmail.com; 2 . Nurse, Ph.D. Adjunct Professor. Email: daiamoder@gmail.com https://orcid.org/0000-0003-2376-6474 Universidade Federal do Rio Grande do Sul Brazil daiamoder@gmail.com; 3 . Occupational Safety Engineer, Master. Email: marceladaoud@yahoo.com.br https://orcid.org/0000-0002-1725-4977 Universidade Federal do Rio Grande do Sul Brazil marceladaoud@yahoo.com.br; 4 . Nurse, Ph.D. Associate Professor. Email: giovanacalcagno@furg.br. https://orcid.org/0000-0002-2464-1537 Universidade Federal do Rio Grande do Sul Brazil giovanacalcagno@furg.br; 5 . Nurse, Ph.D. Adjunct Professor. Email: elianeraquelr@yahoo.com.br. https://orcid.org/0000-0003-1626-5698 Universidade Federal de Santa Maria Brazil elianeraquelr@yahoo.com.br; 6 . Nurse. Email: francielesoares933@gmail.com. https://orcid.org/0000-0003-4356-8416 Universidade Federal do Rio Grande do Sul Brazil francielesoares933@gmail.com; 7 . Nurse, Ph.D. Full Professor. Email: lucianolourencao.enf@gmail.com. https://orcid.org/0000-0002-1240-4702 Universidade Federal do Rio Grande do Sul Brazil lucianolourencao.enf@gmail.com; 8 . School of Nursing, Federal University of Rio Grande, Rio Grande, Rio Grande do Sul, Brazil. Universidade Federal do Rio Grande do Sul School of Nursing Federal University of Rio Grande Rio Grande Rio Grande do Sul Brazil; 9 . Department of Nursing, Federal University of Santa Maria. Santa Maria, Rio Grande do Sul, Brazil. Universidade Federal de Santa Maria Department of Nursing Federal University of Santa Maria Santa Maria Rio Grande do Sul Brazil

**Keywords:** neck pain, quality of life, health professionals, intensive care units, occupational health., dolor de cuello, calidad de vida, personal de salud, unidades de cuidados intensivos, salud laboral, dor cervical, qualidade de vida, profissionais de saúde, unidades de terapia intensiva, saúde ocupacional.

## Abstract

**Objective.:**

To analyze the repercussions of neck pain on the quality of life of health professionals in intensive care units.

**Methods.:**

Cross-sectional, descriptive and correlational study, carried out with 94 health professionals (21 nurses, 13 physical therapists and 60 nursing technicians) in Intensive Care Units of two medium-sized hospitals in a municipality in the far south of Brazil. An instrument containing variables of sociodemographic and work environment characterization was applied; the Neck Bournemouth Questionnaire *(*NBQ*)* and the WHOQOL-Bref were applied.

**Results.:**

There was a predominance of female professionals (88.3%), white (78.8%), aged 30 to 39 years (34.1%), with family income between one and two minimum wages (31.9%) and weekly workload between 31 and 40 hours (67%), night shift (54.3%), time of professional experience of one to five years (38.3%) and one job (73.4%). Neck pain and disability showed significant negative correlations with quality of life. The relationship was weak with the physical (r: -0.218; *p*=0.035) and psychological (r: -0.280; *p*=0.006) domains, and moderate with social relationships (r: -0.419; *p*<0.001), environment (r: -0.280; *p*<0.001) and general quality of life (r: -0.280; *p*<0.001). Overall quality of life showed a moderate correlation with the feeling of anxiety (r: -0.431; p<0.001) and depression (r: -0.515; *p*<0.001) of professionals in the last week.

**Conclusion.:**

Neck pain caused repercussions in the physical, psychological, social, environmental and general quality of life of health professionals in intensive care units.

## Resumen

**Objetivo.:**

Analizar las repercusiones del dolor cervical en la calidad de vida de los profesionales sanitarios de las unidades de cuidados intensivos**.**

**Métodos.:**

Estudio transversal, descriptivo y correlacional de 94 profesionales de la salud (21 enfermeros, 13 fisioterapeutas y 60 técnicos de enfermería) de las unidades de cuidados intensivos de dos hospitales de tamaño medio de un municipio del extremo sur de Brasil. Se aplicó un instrumento que contenía variables sociodemográficas y de caracterización del ambiente de trabajo; el Cuestionario Neck Bournemouth (NBQ) y el WHOQOL-Bref.

**Resultados.:**

Hubo predominio de profesionales del sexo femenino (88.3%), raza blanca (78.8%), edad entre 30 y 39 años (34.1%), con renta familiar entre uno y dos salarios mínimos (31.9%); una carga horaria semanal entre 31 y 40 horas (67%), turno nocturno (54.3%) y años de trabajo entre uno y cinco años (38.3%) y tienen un solo empleo (73.4%). El dolor cervical y las incapacidades laborales mostraron correlaciones negativas significativas con la calidad de vida: la relación fue débil con los dominios físico (r: -0.218; *p*=0.035) y psicológico (r: -0.280; *p*=0.006), y moderada con las relaciones sociales (r: -0.419; *p*<0.001), el entorno (r: -0.280; *p*<0.001) y la calidad de vida general (r: -0.280; *p*<0.001). La calidad de vida global mostró una correlación moderada con los sentimientos de ansiedad (r: -0.431; p<0.001) y depresión (r: -0.515; *p*<0.001) de los profesionales en la última semana.

**Conclusión.:**

El dolor cervical repercute en la calidad de vida física, psicológica, social, ambiental y general de los profesionales sanitarios de las unidades de cuidados intensivos.

## Resumo

**Objetivo.:**

Analisar as repercussões da dor cervical na qualidade de vida de profissionais de saúde de unidades de terapia intensiva.

**Métodos.:**

Estudo transversal, descritivo e correlacional, realizado com 94 profissionais de saúde (21 enfermeiros, 13 fisioterapeutas e 60 técnicos em enfermagem) em Unidades de Terapia Intensiva de dois hospitais de médio porte de um município do extremo sul do Brasil. Foi aplicado um instrumento contendo variáveis de caracterização sociodemográfica e do ambiente de trabalho; o *Neck Bournemouth Questionnaire (*NBQ*)* e o WHOQOL-Bref.

**Resultados.:**

Houve predomínio de profissionais do sexo feminino (88.3%), brancos (78.8%), na faixa etária de 30 a 39 anos (34.1%), com renda familiar entre um e dois salários-mínimos (31.9%) e carga horária de trabalho semanal entre 31 e 40 horas (67%), turno de trabalho noturno (54.3%), tempo de atuação profissional de um a cinco anos (38.3%) e um emprego (73.4%). A dor cervical e incapacidade mostraram correlações negativas significativas com a qualidade de vida. A relação foi fraca com os domínios físico (r: -0.218; *p*=0.035) e psicológico (r: -0.280; *p*=0.006), e moderada com relações sociais (r: -0.419; *p*<0.001), meio ambiente (r: -0.280; *p*<0.001) e qualidade de vida geral (r: -0.280; *p*<0.001). A qualidade de vida geral apresentou correlação moderada com o sentimento de ansiedade (r: -0.431; p<0.001) e de depressão (r: -0.515; *p*<0.001) dos profissionais, na última semana.

**Conclusão.:**

A dor cervical causou repercussões nos domínios físico, psicológico, social, meio ambiente e na qualidade de vida geral dos profissionais de saúde das unidades de terapia intensiva.

## Introduction

Neck pain is a multifactorial condition, which proves to be a public health problem of modern society, especially of health professionals, who deal with the pain of patients, often neglecting their own pain. Although it is not the most prevalent disorder among the population, it is usually in great demand and has a direct impact on work and productivity, in addition to life outside the work environment.^1^ According to the Global Burden of Disease (GBD) study, neck pain was classified as the third reason for years lived with disability among the young population, aged between 20 and 24 years.^2^ A review study showed that psychosocial factors involving stress, anxiety, depression, kinesiophobia, low satisfaction and high work overload can negatively influence neck pain.^1^ As it is a complex and recurrent disorder, it has a strong tendency to chronicity, tending to generate pain, limitation of activities of daily living, incapacity for work activities and reduced Quality of Life (QoL).^3^^,^^4^

Quality of life can be defined as the individual's perception of his position in life, in the context of the culture and value system in which he lives, and in relation to his goals, expectations, standards and concerns, and can be evaluated in various ways.^5^^,^^6^ Among the various QoL assessment instruments, the abbreviated version of the WHOQOL-Bref has been widely used, as it is easy to apply and addresses physical, psychological, social and environmental aspects, as well as general quality of life.^5^ The main causes of pain among health professionals are occupational etiology, resulting from movements of the upper limbs at inadequate angulations, excessive and compensatory efforts.^1^^,^^7^^,^^8^ Neck pain caused by musculoskeletal disorders related to the work environment, such as repetitive movements and neck maintenance in static postures, for long periods of time can interfere with the quality of life of these professionals.^1^

It is common for health professionals in Intensive Care Units (ICU) to face high physical and psychological work demands, resulting from the physical deficiencies of the work environment, the lack of Personal Protective Equipment (PPE) and the lack of professionals qualified for the job.^9^ Dealing with issues of anxiety, depression and post-traumatic stress during the pandemic is a fact linked to neck pain, in addition to being a factor that can trigger them.^1^^,^^10^ In addition, studies show that neck pain arising from the work process was potentiated during the COVID-19 pandemic period, which contributed to the increase in psychological disorders in the workplace.^1^^,^^9^^,^^10^ Although there are studies on neck pain, quality of life and work environment, the approach does not occur in an integrated way, generating a gap in knowledge about the repercussions of neck painful processes on quality of life and its relationship with the work environment in health professionals of Intensive Care Units. In this context, the research question arose: What is the relationship of neck pain in the QoL of professionals working in intensive care units? To answer it, the objective of the study was established: to analyze the repercussions of neck pain on the quality of life of health professionals in intensive care units.

## Methods

This is a cross-sectional, descriptive and correlational study, from the macroproject "Work process and health of clinical-occupational workers in different socio-environmental contexts and age groups", developed between April and August 2023, in Intensive Care Units of two medium-sized hospitals in a municipality in the far south of Brazil, being: (i) a university hospital, which has an adult ICU composed of six beds intended only for the care of patients in the public health system, the Unified Health System (SUS); (ii) a philanthropic hospital, which mostly serves patients from the SUS, but provides care for private patients and the supplementary health network (medical insurance). This hospital has a general ICU with seven intensive and three semi-intensive beds, for SUS care; a Postoperative Intensive Unit with nine beds, for the care of patients in the postoperative period of cardiac surgery and general cardiological care; and a general ICU with ten beds, for the care of private and affiliated patients. 

The study population consisted of nurses, physical therapists or nursing technicians from therapy units who had been working in health institutions for at least two months, which is the average period of adaptation of workers to organizational dynamics.^11^ Professionals who, even meeting the inclusion criteria, were on vacation or on sick leave during the data collection period were excluded from the study. The population eligible for the study consisted of 124 professionals: 29 nurses (10 from the university hospital; 19 from the philanthropic hospital), 73 nursing technicians (21 from the university hospital; 52 from the philanthropic hospital) and 22 physical therapists (7 from the university hospital; 15 from the philanthropic hospital). All eligible professionals were invited to participate in the study and the sample was constituted by convenience. However, to ensure the representativeness of the professionals, the sample size was calculated using StatCalc Epi Info version 7.2. The 95% confidence level and the 5% margin of error were adopted, obtaining the sample size of 94 professionals.

For data collection, carried out from April to August 2023, a self-administered instrument composed of three parts was used, being: a) a sociodemographic questionnaire and characteristics of the work environment*:* built based on studies by the Reference Center for Occupational Health of Bahia de Ilha Grande.^12^ This questionnaire was composed of independent variables (professional category, sex, age, skin color, family income, work shift, hours worked, time of professional experience, number of jobs and if you have ever suffered an accident at work) and characteristics of the work environment (professionals' assessment of the volume of work; and conditions of furniture, equipment and physical space); b) the Neck Bournemouth Questionnaire (NBQ), used to assess aspects related to the level of pain and cervical disability. The version validated in Brazil by Kamonseki *et al.*^13^ has seven questions, with answers on a numerical scale ranging from zero (lowest intensity) to 10 (maximum intensity). The seven questions of the instrument are distributed to score the level of neck pain (NBQ1), how much pain impaired daily activities (NBQ2), the impairment of recreational and leisure activities (NBQ3), feelings of anxiety, tension, irritability (NBQ4), feeling of sadness and depression (NBQ5), worsening of neck pain during activities in the last week (NBQ6) and the way the participant managed pain properly (NBQ7); and c) the WHOQOL-bref, validated in Brazil by Fleck^14^ and used to assess quality of life. The instrument presents 26 questions, with answers on a five-point Likert scale, which comprise five domains: physical (covers pain and discomfort, energy and fatigue, sleep and rest, activities of daily life, dependence on medication or treatments, work capacity); psychological (it includes positive feelings, thinking, learning, memory and concentration, self-esteem, body image and appearance, negative feelings, spirituality, religiosity and personal beliefs); social relationships (it involves personal relationships, social support and sexual activity); environment (it considers physical safety and protection, home environment, financial resources, health and social care, recreation/leisure, physical environment), in addition to general quality of life (general QoL).^14^

To verify that the questionnaires did not present possible misunderstandings, a pilot study was carried out with eight professionals from one of the work shifts. As there was no need for semantic and structural reformulation, the professionals who participated in the pilot study were included in the final sample. The application of the research instruments was conducted by a physical therapist with professional experience in the ICU. Professionals were invited to participate in the study through an informative text sent in the ICU WhatsApp groups by the technical managers of each unit. The professionals were also approached in person by the researcher and invited to fill out the study questionnaire. The distribution of the questionnaires was carried out by work shift, in each of the units. Three attempts were made on different days, in order to find the professionals who, perhaps, had not been approached. The professionals answered the questions in the workplace. The mean application time was approximately 30 minutes. In case of unavailability due to complications in the unit, the professionals were allowed to answer the instruments at another time, outside the work environment, with prior guidance from the researcher and the return of the completed instruments at the next visit of the researcher to the unit. In addition, the researcher's contact was made available to clarify doubts. Four professionals formally refused to participate in the study.

The data obtained were tabulated in the Statistical Package for the Social Science (SPSS), version 28.0. The assessment of data normality was performed using the Kolmogorov-Smirnov test. To assess the level of pain and cervical disability, the scores of all questions on the Neck Bournemouth Questionnaire were summed*,* resulting in a value from zero to 70, considering that the higher the score obtained, the higher the level of pain and cervical disability.^13^ To calculate the *WHOQOL-*bref results, the scores for each domain were calculated, considering: (i) physical domain: (Mediax6 (Q3+Q4+Q10+Q15+Q16+Q17+Q18))x4; (ii) psychological domain: Mediax5 (Q5+Q6+Q7+Q11+Q19+Q26))x4; (iii) social relations domain: (Mediax2 (Q20+Q21+Q22))x4; (iv) environmental domain: (Mediax6 (Q8+Q9+Q12+Q13+Q14+Q23+Q24+Q25))x4. Then, the obtained scores were converted to a scale from 0 to 100 using the formula [(Mean - 4) × 100/16]. The higher the scores, the better the assessment of quality of life.^14^

For the descriptive analysis, central tendency measurements (mean and median) and dispersion measurements (standard deviation and interquartile range) were performed, according to data distribution. After describing the absolute and relative frequencies, the means were compared using analysis of variance (ANOVA). To evaluate the correlation between the variables, the *Spearman* Correlation test was applied. The interpretation of the correlation was classified as weak for r values up to 0.399, moderate for values between 0.400 and 0.699, and strong for values equal to or greater than 0.700.^10^ All analysis adopted a significance level of 5%. 

The study was approved by the Institution's Research Ethics Committee under Certificate of Presentation of Ethical Appreciation number 63105722.2.0000.5324. Before data collection, participants were informed about the purpose of the study and signed the Informed Consent Form. 

## Results

The study included 94 health professionals, 28 (29.8%) from the university hospital and 66 (70.2%) from the philanthropic hospital. Among the professionals assessed, 21 (22.4%) were nurses, 60 (63.8%) nursing technicians and 13 (13.8%) physical therapists. As shown in Table 1, there was a predominance of females (88.3%), white skin color (78.8%), age group from 30 to 39 years (34.1%), and family income between one and two minimum wages (31.9%), weekly workload between 31 and 40 hours (67.0%), night shift (54.3%), time of professional experience from one to five years (38.3%) and with only one job (73.4%).

**Table 1:** Sociodemographic and professional characteristics of health workers in Intensive Care Units. Rio Grande, RS, 2023. (*n*=94)

Variables	*n*	%
Work institution		
University Hospital	28	29.8
Philanthropic Hospital	66	70.2
Professional Category		
Nurse	21	22.3
Nursing technician	60	63.8
Physical therapist	13	13.8
Sex		
Male	11	11.7
Female	83	88.3
Skin color		
White	74	78.8
Black and brown	20	21.2
Age group		
20 to 29 years	25	26.6
30 to 39 years	32	34.1
40 to 50 years	31	33.0
Over 50 years	6	6.4
Monthly family income		
1 to 2 minimum wages	30	31.9
2 to 3 minimum wages	22	23.4
3 and ≤ 5 minimum wages	23	24.5
5 and ≤ 20 minimum wages	20	20.2
Weekly workload		
≥ 20 and ≤ 30 hours	18	19.1
≥ 31 and ≤ 40 hours	57	67.0
> 40 hours	13	13.8
Work Shift		
Daytime	43	45.7
Nighttime	51	54.3
Time of experience in the profession		
≤ 12 Months	9	9.6
One to five years	36	38.3
Six to ten years year;	15	16.0
> 10 years	34	36.2
Number of Jobs		
One	96	73.4
Two	21	22.3
Three	4	4.3
Has been through work accidents		
Yes	33	35.1
No	61	64.9

*Minimum wage amount: R$1320.00 ≈ USD 264.05 (1 USD = R$4.9997)

Data on the work environment were described through qualifiers, in which 61.7% of the professionals evaluated that there is a condition of overload in relation to the volume of service. The furniture of the work sector was considered in unsatisfactory conditions (regular, bad or very bad) by 61.7% of the professionals. Although the percentage of professionals who consider work sector equipment as excellent or good (48.9%), for more than 50% of professionals, these equipment have regular or poor conditions (Table 2).

**Table 2:** Characteristics of the work environment of health workers in Intensive Care Units. Rio Grande, RS, 2023. (*n*=94)

Variables	*n*	%
How do you evaluate the volume of work?		
Light	1	1.1
Moderate	35	37.2
There is an overload	49	52.1
Exhaustive	9	9.6
How do you evaluate the furniture in your work sector?		
Excellent	2	2.1
Good	34	36.2
Regular	46	48.9
Bad	11	11.7
Very bad	1	1.1
How do you evaluate the equipment in your work sector?		
Excellent	4	4.2
Good	42	44.7
Regular	42	44.7
Bad	1	1.1

The analysis of the Neck Bournemouth Questionnaire (Table 3) showed that there was no statistical difference in the mean scores obtained by each professional category (*p*>0.05). It is noteworthy, however, that the professionals obtained higher scores in the questions referring to the presence of anxiety (question 4) and neck pain (question 1) in the last week.

**Table 3:** Scores for the questions of the Neck Bournemouth Questionnaire, according to the professional category of workers in the Intensive Care Units. Rio Grande, RS, 2023. (*n*=94)

Scores for the Neck Bournemouth Questionnaire	Nurses	Nursing Technicians	Physical therapists	***p*-value**
	Mean (_95%_ CI)	Mean (_95%_ CI)	Mean (_95%_ CI)	
1. What was your level of neck pain	5.3 (4.1 - 6.5)	4.3 (3.6 - 5.1)	3.6 (1.8 - 5.4)	0.198
2. How much did your neck pain impair your daily activities (Homework, bathing, putting on clothes, getting up, reading and driving)	4.1 (2.8 - 3.8)	3.0 (2.3 - 3.8)	2.8 (0.9 - 4.7)	0.307
3. How much did your neck pain affect your recreational, social and family activities?	3.6 (2.5 - 4.8)	2.7 (2.0 - 3.4)	2.1 (0.3 - 3.9)	0.232
4. Did you feel anxious (tense, nervous, irritable, having difficulty concentrating/relaxing)?	6.3 (4.9 - 7.6)	6.1 (5.3 - 6.9)	4.8 (2.8 - 6.8)	0.317
5. Did you feel depressed ("down", sad, pessimistic, and unhappy)?	4.6 (3.0 - 6.1)	4.4 (3.5 - 5.3)	3.8 (1.8 - 5.7)	0.794
6. How much has your neck pain gotten worse, (or could it have gotten worse) with work both outside and at home?	4.5 (3.3 - 5.8)	3.4 (2.6 - 4.1)	3.2 (1.0 - 5.5)	0.283
7. How much have you been able to control (reduce) your neck pain on your own?	3.9 (2.8 - 5.0)	3.1 (2.4 - 3.7)	1.3 (0.5 - 3.3)	0.079

**Figure 1:**
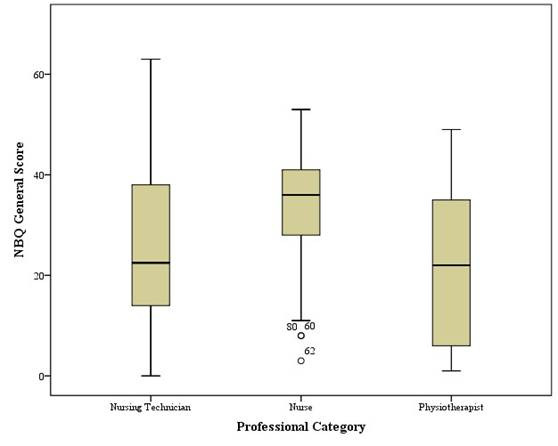
General score of the Neck Bournemouth Questionnaire, according to the professional category of workers in the Intensive Care Units. Rio Grande, RS, 2023. (*n*=94)

The evaluation of the level of pain and cervical disability, based on the analysis of the NBQ score in percentiles, revealed that 27 (28.7%) professionals had a score ≤ 14 points (1^st^ percentile); 43 (54.8%) had a score > 14 and < 75 points (2^nd^ percentile); and 24 (25.5%) professionals had a score ≥ 75 points (3^rd^ percentile). The latter are the ones with the highest level of pain and cervical disability, being: 7 (29.2%) nurses, 14 (58.3%) nursing technicians and 3 (12.5%) physical therapists. Figure 1 shows the analysis of the overall NBQ score, by professional category. 

Regarding the evaluation of quality of life, the professionals had a lower score in the physical domain (55.2 points) and a higher score in the social relationships domain (68.1points). Nurses had a significantly lower score than other professionals in the physical domain and nursing technicians had a significantly higher score than other professionals for overall quality of life (Table 4).

**Table 4:** Mean WHOQOL-Bref scores of workers in Intensive Care Units. Rio Grande, RS, 2023. (n=94)

WHOQOL-bref DOMAINS	Nurses	Nursing Technicians	Physical therapists	Total	***p*-value**
	Mean (_95%_ CI)	Mean (_95%_ CI)	Mean (_95%_ CI)	Mean (_95%_ CI)	
Physical	50.9 (46.8 - 54.9)	57.3 (54.9 - 59.6)	52.7 (47.7 - 57.8)	55.2 (47.7 - 57.8)	0.010
Psychological	60.1 (56.4 - 63.8)	63.0 (59.9 - 66.0)	61.9 (54.7 - 69.0)	62.2 (59.9 - 64.5)	0.595
Social Relationships	63.9 (57.1 - 70.6)	68.8 (64.0 - 73.5)	71.8 (57.8 - 85.8)	68.1 (64.3 - 71.9)	0.435
Environment	52.5 (46.7 - 58.4)	61.2 (58.1 - 64.3)	58.9 (47.8 - 70.0)	58.9 (56.2 - 61.7)	0.039
Overall QoL	55.4 (52.6 - 58.2)	61.4 (59.0 - 63.9)	59.5 (52.3 - 66.6)	59.8 (57.9 - 61.8)	0.037

As shown in Table 5, there was a negative and statistically significant correlation of neck pain and disability with quality of life. There was a weak correlation between neck pain and disability and the physical (r: -0.218; *p*=0.035) and psychological (r: -0.280; *p*=0.006) domains of quality of life; and moderate correlation of neck pain and disability with the social relationships (r: -0.419; *p*<0.001) and environment (r: -0.280; *p*<0.001) and with overall quality of life (r: -0.280; *p*<0.001) domains. It is also noteworthy the moderate correlation of general quality of life with the feeling of anxiety (r: -0.431; *p*<0.001) and depression (r: -0.515; *p*<0.001) of professionals during the last week.

**Table 5:** Correlations between Neck Pain and Disability (NBQ) with WHOQOL-BREF DOMAINS

NBQ Questions	WHOQOL-BREF DOMAINS			
	Physical	Psychological	Social Relationships	Environment	Overall QoL
1. During the last week, what was your level of neck pain	-0.220^*a^ (0.033)	-0.095^a^ (0.362)	-0.259^*a^ (0.012)	-0.222^*a^ (0.032)	-0.242^*a^ (0.019)
2. During the last week, how much has your neck pain hindered your daily activities (Homework, bathing, putting on clothes, get up, reading and driving)	-0.167^a^ (0.107)	-0.244^*a^ (0.018)	-0.315^**a^ (0.002)	-0.363^**a^ (<0.001)	-0.351^**a^ (0.001)
3. During the last week, how much did your neck pain affect your recreational, social and family activities?	-0.166^a^ (0.110)	-0.212^*a^ (0.040)	-0.343^**a^ (0.001)	-0.394^**a^ (<0.001)	-0.357^**a^ (<0.001)
4. During the last week, have you felt anxious (tense, nervous, irritable, having difficulty concentrating/relaxing)?	-0.220^*a^ (0.033)	-0.346^**a^ (0.001)	-0.339^**a^ (0.001)	-0.380^**a^ (<0.001)	-0.431^**b^ (<0.001)
5. During the last week, have you felt depressed ("down", sad, pessimistic, unhappy)?	-0.222^*a^ (0.031)	-0.449^**b^ (<0.001)	-0.464^**b^ (<0.001)	-0.432^**b^ (<0.001)	-0.515^**b^ (<0.001)
6. During the last week, how much has your neck pain gotten worse, (or could it have gotten worse) with work both outside and at home?	-0.152a (0.144)	-0.197^a^ (0.057)	-0.346^**a^ (0.001)	-0.358^**a^ ((<0.001)	-0.339^**a^ (0.001)
7. During the last week, how much have you been able to control (reduce) your neck pain on your own?	-0.103^a^ (0.323)	0.003^a^ (0.980)	-0.269^**a^ (0.009)	-0.241^*a^ (0.019)	-0.197^a^ (0.057)
Total Score	-0.218^*a^ (0.035)	-0.280^**a^ (0.006)	-0.419^**b^ (<0.001)	-0.420^**b^ (<0.001)	-0.431^**b^ (<0.001)

* p<0.05. **p<0.01. ^*a*^ *Weak correlation;* ^*b*^ *Moderate correlation.*

## Discussion

The study made it possible to analyze the work environment of health professionals in Intensive Care Units, examining how neck pain affects the quality of life of these workers. The results showed that neck pain has the potential to negatively impact the quality of life of the health professionals studied. Musculoskeletal pain, especially neck pain accompanied by disability, usually originates in the work environment and affects several aspects of health. They negatively impact the physical and mental health and overall quality of life of health professionals.^4^^,^^15^ Nevertheless, during the pandemic period there was a mix of feelings capable of enhancing existing pains, due to the environment and work routine, or contributing to the emergence of new pains.

Workers have a higher risk of developing neck pain when facing high work demands.^7^^,^^16^ As shown in the present study, the greater the volume of work felt by workers, the greater the perception of neck pain. In this sense, it is important to highlight that the units studied have different characteristics. Some attend a high turnover of patients, which can increase the physical wear of professionals. Other units deal with patients with chronic diseases, which generates additional physical and psychological demands for workers. In addition, some services focus on the conduct established only by physicians, disregarding the contribution of other professionals. The literature points to the lack of support from colleagues and the limitation in decision-making power as risk factors for the onset of neck pain.^7^^,^^16^

Another point to be highlighted is the repercussion that the volume of service perceived by workers causes in activities of daily living, leisure, social or family activities. The intensity of low back pain is associated with worse prognosis for pain relief and greater physical limitations for daily and social activities.^17^ Nevertheless, neck and lower back pain can be influenced by work strain caused by work-life imbalance. The constant pressure to follow conducts that disregard multidisciplinary collaboration can generate tensions. When these stresses are combined with low pay and the need to work more than one job, professionals are more vulnerable to musculoskeletal problems. Therefore, strategies that reduce tensions in the health team can be effective in preventing these injuries.^1^^,^^17^ A study with Primary Health Care professionals indicated that neck pain affected only the physical and psychological domains of quality of life.^18^ In contrast, our study of intensive care unit professionals revealed that the level of neck pain was associated not only with the physical domain but also with the social, environmental, and overall quality of life domains. The association of higher levels of neck pain with lower quality of life scores observed in our study suggests a possible relationship between the complexity of the work environment and the occurrence of neck pain.

A study carried out in China showed that nursing professionals presented, primarily, pain in the back and lower limbs. In addition to estimating that more than a third of professionals reported pain reflexes in their daily lives and sleep impairments.^19^ Similar results were found in the present study, where health professionals who reported greater impact of neck pain on daily activities showed a strong association with the physical domain. This domain includes the evaluation of factors such as pain and discomfort, day-to-day activities, sleep and rest.^14^ Saudi health professionals presented with high-intensity neck pain when compared to symptoms of shoulder pain.^20^ It is common for cervical spine pain to have a negative impact on the physical and mental health of workers. In this sense, psychological factors, such as anxiety, are responsible for worsening neck pain crises, in addition to contributing to disability and kinesiophobia. In addition, patients with neck pain crises may experience periods of anxiety.^1^^,^^21^ In addition to anxiety, problems related to depressive events also contribute to neck pain, creating a bidirectional mechanism in which pain and disability reinforce each other.^1^^,^^21^ Feelings of sadness, pessimism, and unhappiness are often associated with neck pain disorders, which can lead to high morbidity.^1^ However, to diagnose a depressive disorder, it is necessary for the individual to be evaluated by a mental health professional. In addition, the excessive volume of work and the lack of decision-making power make the professional feel powerless, which can result in negative consequences outside the work environment.^18^

Some osteokinematic movements, when performed excessively or sustained by long hours of work, such as cervical flexion and rotation, are associated with psychosomatic symptoms, such as anxiety, depression and Post Traumatic Stress Disorder (PTSD).^20^^,^^22^ As elucidated in our study, the worsening of neck pain during work was a reality found that, in turn, compromised all domains of QoL. A meta-synthesis identified that neck pain proves to be of multidimensional phenomenology, affecting both the physical and psychological domains, as well as the social one.^23^ In the present study, we observed an inverse correlation between the ability to control or reduce neck pain and quality of life, indicating that health professionals who face more difficulty in controlling pain have greater losses in the physical, social, general and environmental domains. According to the literature, there are different strategies for coping with neck pain, such as psychotherapy, pharmacological treatments, electrotherapy, dietary changes, mental health resources, lifestyle adaptations and regular practice of physical activity.^24^

A qualitative study conducted in Canada^24^ showed that chronic pain represents an important factor of interference in all aspects of QoL, in addition to highlighting the importance of a clinical diagnosis, and not only strategies for coping with it. In the case of Brazil, the low remuneration received by professionals leaves them hostage to drug treatments, which are often ineffective because they do not treat the cause. This situation leads to chronic pain, which can culminate in incapacity for work. To face this problem, strategies are needed that promote the adequate sizing of professionals for the demands of health units, the availability of equipment that assists in the manual transport of cargo and the implementation of public policies for valuing and promoting the health of professionals in intensive care units.^6^^,^^25^

It is concluded that neck pain has had repercussions on several aspects of the lives of health professionals working in intensive care units, including the physical, psychological, social, and environmental and quality of life domains. The increase in pain and disability affects feelings of anxiety and depression, further contributing to the worsening of the quality of life and well-being of these professionals.

As a limitation of the study, it is noteworthy that the sample restricted to two hospital units in a municipality in southern Brazil may not capture the diversity of work environments and conditions that influence neck pain and quality of life, resulting in a reduced view of the factors involved and the dynamics of evolution of these conditions. However, the results obtained are able to support the implementation of prevention and intervention strategies that can reduce the incidence of neck pain and its consequences on the quality of life of workers, such as ergonomics and psychological support programs. In addition to directing institutional managers and professionals responsible for occupational health to make changes in the work environment and in worker support practices, aiming to improve the quality of life and well-being of these 
